# Efficacy of an exercise oncology program on sleep quality and circadian rhythm in head and neck cancer patients undergoing chemo-radiation therapy: a randomized controlled trial

**DOI:** 10.7717/peerj.21409

**Published:** 2026-06-25

**Authors:** Cherishma Dsilva, Vijith V. Shetty, Donald Fernandes, Jean Pierre Baeyens, Suchetha Kumari N., Saumya Srivastava, Stephen Rajan Samuel

**Affiliations:** 1 Nitte (Deemed to be University), Nitte Institute of Physiotherapy (NIPT), Mangalore, Karnataka, India; 2 Father Muller College of Physiotherapy, Father Muller Charitable Institutions, Mangalore, Karnataka, India; 3 Nitte (Deemed to be University), KS Hegde Medical Academy (KSHEMA), Department of Medical Oncology, Mangalore, Karnataka, India; 4 Department of Radiation Oncology, Father Muller Medical College Hospital, Mangalore, Karnataka, India; 5 THIM Internationale Hochschule für Physiotherapie, Landquart, Switzerland; 6 Srinivas Institute of Medical Sciences and Research Center, Srinivas University, India; 7 Division of Health and Life Sciences, College of Arts & Sciences, Mount Vernon Nazarene University, Ohio, United States of America

**Keywords:** Head and neck cancer, Chemo-radiotherapy, Sleep quality, Circadian rhythm, Melatonin, Exercise oncology, Urinary melatonin, Exercise

## Abstract

**Background:**

Head and neck cancer (HNC) presents a significant challenge in oncology. It has high prevalence rates, especially in India, where 30–40% of cancer cases are attributed to HNC. While chemotherapy and radiation therapy are essential components of treatment, they often lead to significant sleep disturbances that negatively impact patients’ quality of life and the effectiveness of their treatment. These sleep disturbances are frequently associated with disruptions in the circadian rhythm, which are commonly seen in cancer survivors. This study aimed to evaluate the effect of an exercise oncology program on sleep quality and circadian rhythm in patients with HNC undergoing chemo-radiation therapy.

**Methods:**

Patients with HNC (stage III, IVa, or IVb) undergoing chemo-radiation therapy, aged 18 years or above, of any gender, and with an Eastern Cooperative Oncology Group score < 2 were included. Patients with severe orthopedic or neurological conditions, hemoglobin < 8 g/dL, platelet count < 30,000/µL, or unfit for exercise were excluded. Seventy HNC patients were randomized to exercise oncology and control group. The exercise oncology group received 15–20-minute sessions of aerobic (brisk walking) and resistance training (upper and lower limb exercises). Participants in the control group were asked to follow the walking protocol recommended by National Comprehensive Cancer Network guidelines, five days a week for seven weeks. Pittsburgh Sleep Quality Index (PSQI) was used to assess overall sleep quality, while melatonin excretion in urine, quantified using ELISA, was used to evaluate the circadian rhythm. Between-group comparisons were analyzed using mixed analysis of variance for PSQI and analysis of covariance for urinary melatonin. Receiver operating characteristic (ROC) curve analysis was used to evaluate the diagnostic accuracy of PSQI scores and melatonin levels.

**Results:**

PSQI significantly improved in the exercise group (13.97 ± 2.29 to 7.09 ± 1.15), and significantly worsened in controls, (13.06 ± 1.73 to 14.14 ± 2.64) (both *p* < 0.001). Although urinary melatonin levels increased in the exercise group (284.16 ± 85.29 ng/L to 369.75 ± 91.61 ng/L) and decreased in controls (368.00 ± 100.62 ng/L to 304.39 ± 99.50 ng/L), the between-group difference was not significant after adjusting for baseline values (*p* = 0.372). Furthermore, ROC curve analysis demonstrated that post-intervention PSQI exhibited excellent diagnostic accuracy for distinguishing between good and poor sleep quality.

**Conclusion:**

An exercise oncology program has potential benefits in improving quality of sleep and regulating the circadian rhythm in HNC patients undergoing chemo-radiation therapy.

## Introduction

Head and neck cancer (HNC) is one of the most debilitating malignancies in oncology, encompassing a wide range of tumors that originate from the mouth, throat, larynx, and surrounding tissues (Johnson et al., 2020). According to the GLOBSCAN 2018 report, HNC ranks among the most prevalent cancers globally with an annual incidence of 834,860 cases and 431,131 deaths (Elkashty, Ashry & Tran, 2019; Khetan et al., 2019). In developing countries, including India, HNC present unique challenges for healthcare systems. They account for nearly 30–40% of all cancer cases and therefore represent a major contributor to the overall cancer burden in the population (Khetan et al., 2019).

For cases of HNC that are locally advanced, concurrent chemo-radiation has become the standard treatment approach, with the potential to cure (Reyes et al., 2023). While this combined modality helps to suppress tumor progression and limit the risk of metastasis, it is also accompanied by substantial physical and psychological adverse effects (Anderson et al., 2021; Yeh, 2010). Poor sleep quality in cancer patients has been demonstrated to negatively impact mental health, reduce work productivity, lower quality of life, increase healthcare utilization, and predict other complications (Amjad, Chidharla & Kasi, 2024; Was et al., 2022; Bhutani et al., 2024; Lee et al., 2023). Patients with HNC experience significant sleep disruption during and following treatment, exacerbating fatigue and negatively impacting their quality of life (Amjad, Chidharla & Kasi, 2024; Supportive & Palliative Care Editorial Board, 2002). Consequently, evaluating sleep quality is essential to improving overall health outcomes and potentially enhancing treatment response (Stein, Syrjala & Andrykowski, 2008; Anjanappa et al., 2020). Focusing on sleep quality allows healthcare providers to deliver more holistic care, ultimately supporting both the physical recovery and psychological well-being of patients (Amjad, Chidharla & Kasi, 2024).

Dependable measures of sleep are essential for identifying and managing disturbances within individuals with HNC. The Pittsburgh Sleep Quality Index (PSQI) is widely used to evaluate sleep quality in cancer patients (Bergamaschi et al., 2023; Thureau et al., 2021). Additionally, measuring melatonin excretion in urine serves as an objective biomarker of sleep quality (Hua et al., 2020). Melatonin, a hormone secreted by the pineal gland, is integral to regulating the sleep-wake cycle. Beyond its sleep-inducing properties, melatonin acts as an antioxidant by inhibiting certain cancer cells, boosting the immune system, alleviating symptoms of depression, and mitigating sleep disturbances caused by factors like shift work or jet lag (Ganju et al., 2019; Chauhan et al., 2020). These indicators have been used in several exercise oncology clinical trials aimed at enhancing treatment efficacy and improving the quality of life for HNC patients undergoing chemo-radiation therapy (Roscoe et al., 2022; Capozzi et al., 2016; Samuel et al., 2019).

Exercise oncology is an emerging field that investigates the role of structured physical activity across the cancer continuum. Evidence suggests that exercise not only alleviates treatment-related symptoms but may also enhance treatment efficacy, modulate tumour biology, delay progression, and improve survival (Lonkvist et al., 2017). Recent studies specifically in HNC populations demonstrate that supervised exercise interventions can improve physical function, mitigate treatment-related side effects, and enhance overall quality of life (Midgley et al., 2018; Perez et al., 2023; Demurtas et al., 2024). These observations provide a strong basis for exploring how exercise might help alleviate sleep problems in individuals undergoing chemo-radiation therapy for HNC.

A randomized controlled trial was conducted to evaluate the efficacy of an exercise oncology program in enhancing sleep quality and circadian rhythm in this population. The findings are expected to contribute to the design of targeted interventions and offer guidance for clinical practice, ultimately supporting more comprehensive care for patients with HNC.

## Material and Methods

### Study design and setting

This randomized, parallel-group trial investigated the impact of a structured exercise oncology program on sleep quality in patients with HNC, specifically those with stages III, IVA, and IVB receiving concurrent chemo-radiation therapy. Participants were enrolled using simple random sampling. This study was conducted at Father Muller Medical College Hospital, Mangalore between November 2023 and November 2024. The study was registered with the Clinical Trials Registry- India (CTRI/2023/11/059839).

### Sample size

The sample size was calculated based on pilot study data, with an expected mean difference of 2.01 points in PSQI and a standard deviation of 3.10 corresponding to Cohen’s d ≈ 0.67. Using a two-tailed test with 95% confidence interval and 80% power, the required sample size was estimated as 35 per group.

### Ethical considerations

The study received approval from the Father Muller Institutional Ethics Committee (FMIEC) (FMIEC/CCM/616/2023), and participants were informed about the study’s objectives. They were screened for inclusion using the following eligibility criteria: patients with HNC (stages III, IVA and IVB) receiving chemo-radiation therapy, with an Eastern Cooperative Oncology Group (ECOG) score of less than 2, aged 18 years and older, and of either gender, along with pre-operative or post-operative status. Written informed consent was obtained from each participant. Patients who were not fit for exercise training, had a platelet count below 30,000/µL, had a hemoglobin count below 8 g/dL, or had severe orthopaedic or neurological issues were excluded.

### Randomization

**Figure 1:**
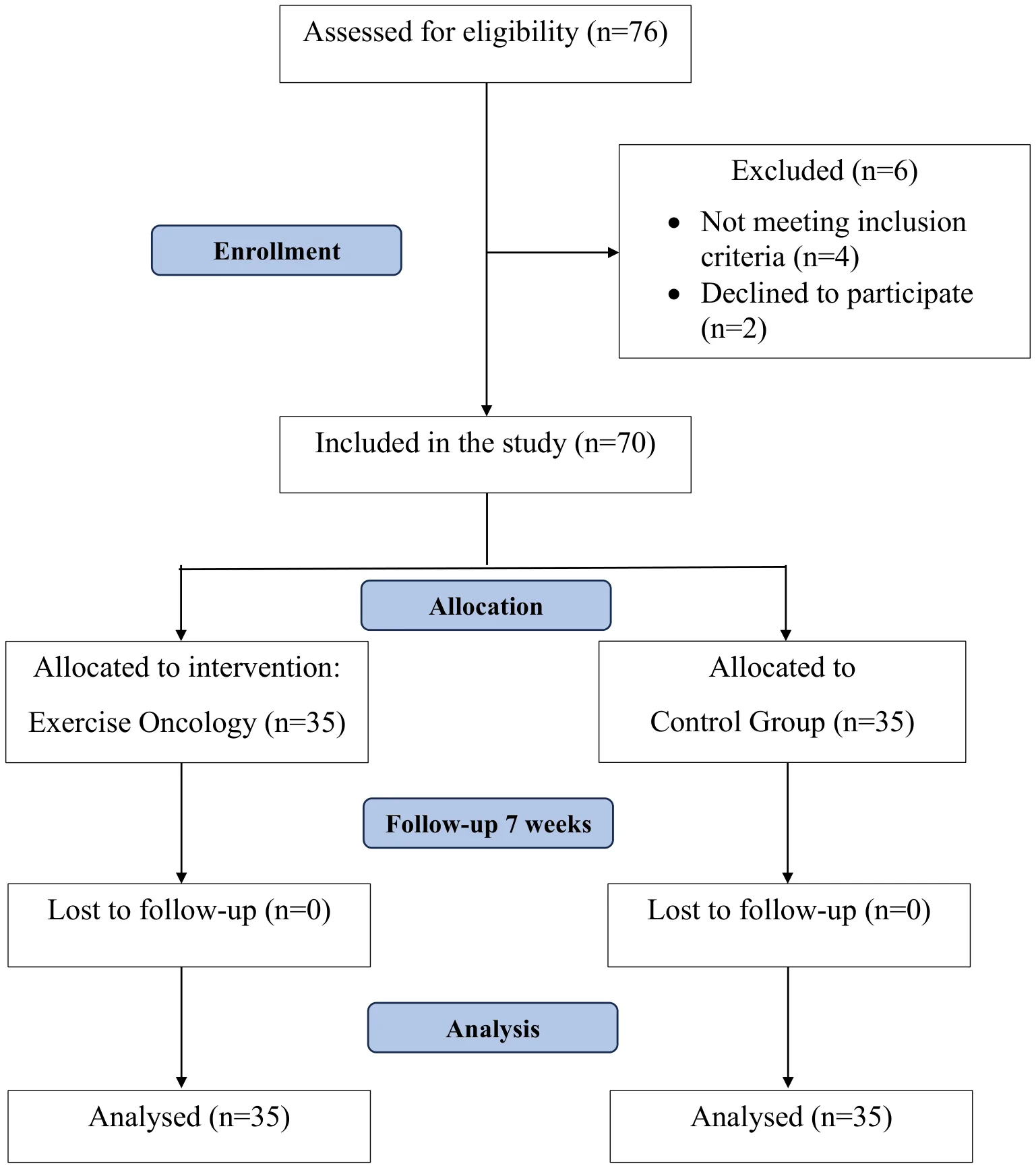
CONSORT flow diagram for the study.

The investigator randomly assigned participants in a 1:1 allocation ratio to either the Intervention group (Group A) or Control group (Group B). The randomization sequence was generated by a statistician using a computer-based random number generator. Block randomization was used to ensure balanced allocation. This study followed Consolidated Standards of Reporting Trials (CONSORT) guidelines (Schulz, Altman & Moher, 2010) as depicted in Fig. 1.

### Blinding

In this study, blinding of participants was not feasible due to the nature of the intervention. The investigator who administered the intervention was also responsible for outcome assessments and was therefore aware of group allocation. However, to minimize potential bias, standardized protocols were strictly followed, and validated, objective tools were used for outcome measurement.

### Intervention

#### Exercise oncology group

For a duration of seven weeks, the exercise oncology group underwent an exercise intervention designed in accordance with the American College of Sports Medicine (ACSM) guidelines and the protocol by anonymous (2010) and Mustian et al. (2012). Participants engaged in supervised exercise sessions five days a week, excluding the day of chemo-radiation therapy. Each session lasted 15–20 min and consisted of both aerobic and resistance components. Aerobic training involved brisk walking, while resistance training targeted the major upper and lower limb muscle groups. Exercises included biceps curls, triceps extension, overhead shoulder flexion, hip flexion, knee extension, and hip abduction performed as two sets of eight to 15 repetitions each using elastic resistance band (red or yellow Theraband^®^, corresponding to light-to-moderate resistance). Resistance exercise intensity was prescribed and adjusted using the OMNI-Resistance Exercise Scale (Robertson et al., 2003), with a target perceived exertion range of 3–5 for each resistance exercise. Overall session exertion was also monitored using the Modified Borg’s Scale of Perceived Exertion (RPE) scale (Parent-Roberge et al., 2020) to ensure patient safety and adherence to moderate-intensity exercise. To minimize variability, all sessions were scheduled between 10:00 am and 12:00 pm, prior to the participant’s chemo-radiation therapy appointment, and this schedule was maintained throughout the intervention period (Demurtas et al., 2024).

#### Control group

Participants in the control group received standard medical care along with advice to walk for 10 min, three times daily, totaling 150 min per week as recommended by the National Comprehensive Cancer Network (NCCN) guidelines (Berger, Fernandez-Robles & Murphy, 2018). Participants in this group were urged to continue being as active as they could.

### Follow up

Throughout the seven-week exercise training period in the hospital during chemo-radiation therapy, the investigator closely monitored participants in both groups on a daily basis for any potential complications.

### Outcome measures

#### Pittsburgh Sleep Quality Index

The seven domains that make up the PSQI questionnaire were administered. A PSQI total score below five denoted high-quality sleep, and a score above five denoted low-quality sleep (Hinz et al., 2025; Al Maqbali et al., 2020).

#### Urinary melatonin

The first morning urine was collected and the Elabscience^®^ Enzyme-Linked Immunosorbent Assay (ELISA) Kit was used to analyze urinary melatonin excretion as an indicator of circadian rhythm changes (Benloucif et al., 2008; Wang et al., 2014; Cook et al., 2002). To reduce inter-individual variability, participants were instructed to avoid caffeine, alcohol, and medications known to influence melatonin secretion for at least 24 h prior to sample collection. All samples were collected at a consistent time, between 6:00 and 7:00 a.m, following an overnight fast. Samples were immediately stored at −80 °C until analysis to preserve stability.

The outcome measures were assessed at two time points: on day 1 of pre chemo-radiation therapy and at the end of the seven-week period. All participants received a standardized chemo-radiation therapy protocol consisting of chemotherapy (Cisplatin 35 mg/m^2^ once a week for 7 weeks) and radiation therapy (70gy in 35 daily fractions for 5 days per week for 7 weeks as per hospital protocol). Melatonin assessments were conducted prior to the initiation of each cycle/at baseline and after completion of 7 weeks, ensuring consistency across patients.

### Statistical analysis

Data were analyzed on an intention-to-treat basis, including all randomized participants. PSQI and melatonin levels were analyzed using outcome-specific models based on baseline comparability. Independent samples t-tests or Mann–Whitney U tests were first used to compare baseline group differences, depending on normality (Table 1). For PSQI, baseline scores did not differ significantly between groups (*p* = 0.064), so a mixed analysis of variance (ANOVA) was conducted with time (pre, post) as the within-subject factor and group (intervention, control) as the between-subject factor. For melatonin, baseline values differed significantly between groups (*p* < 0.001); therefore, an analysis of covariance (ANCOVA) was performed with post-intervention melatonin as the dependent variable and baseline melatonin as a covariate. Model assumptions were checked using Shapiro–Wilk tests for normality and Levene’s/Brown–Forsythe tests for homogeneity of variances. As age was not normally distributed, between-group comparisons were performed using the Mann–Whitney U test. Homogeneity of variances was evaluated using the Brown–Forsythe test. Categorical variables, including gender, cancer site, and TNM stage, were compared between groups using the chi-square (*χ*^2^) test. Effect sizes were reported as Cohen’s d for t-tests, rank biserial correlation for Mann–Whitney tests, and omega squared (*ω*^2^) for ANOVA/ANCOVA. When significant, *post hoc* pairwise comparisons were performed. For PSQI, a Holm–Bonferroni correction was used to adjust for multiple comparisons. For melatonin, Tukey’s Honestly Significant Difference (HSD) test was applied. Adjusted *p*-values and confidence intervals are reported accordingly. Receiver Operating Characteristic (ROC) curve analysis was conducted to evaluate the diagnostic accuracy of PSQI scores (both PSQI pre and PSQI post) and melatonin levels (both melatonin pre and melatonin post) in distinguishing between patients with good and poor sleep quality. The Area Under the Curve (AUC) was used to quantify the discriminatory ability of these biomarkers, with values closer to 1 indicating higher diagnostic accuracy. The *priori* level for significance was set at *p* < 0.05. The data were analyzed using IBM SPSS Statistics (Version 27; IBM Corp., Armonk, NY, USA).

**Table 1:** Baseline group comparisons for pittsburgh sleep quality index and urinary melatonin.

	**Test**	**Statistic**	***p***	**Effect size**
**PSQI Pre**	Independent samples *t*-test	−1.882	0.064	−0.450
	Mann–Whitney U test	505.500	0.202	0.175
**Melatonin Pre**	Independent samples *t*-test	3.608	<0.001	0.862
	Mann–Whitney U test	867.000	0.002	−0.416

**Notes.**

*p* level of significance PSQI Pittsburgh Sleep Quality Index

For the Student *t*-test, effect size is given by Cohen’s d and for the Mann–Whitney test, effect size is given by the rank biserial correlation.

## Results

A total of 76 participants were screened for eligibility, of whom six were excluded (four did not meet the inclusion criteria, and two declined to participate). Seventy participants were ultimately enrolled and randomized equally into Group A (Exercise Oncology; *n* = 35) and Group B (Control; *n* = 35). Figure 1 illustrates the study flow. Baseline demographic and clinical characteristics are presented in Table 2. The majority of participants were male (82.86% in Group A; 89.66% in Group B). Most participants were in cancer stage III or IVA, with the tongue, buccal mucosa, and larynx being the most common cancer sites across groups. Age deviated significantly from a normal distribution (Shapiro–Wilk *p* < 0.001) and equality of variances was confirmed using the Brown–Forsythe test (*p* = 0.850). No significant difference in age was found between groups (*p* = 0.108). There were no statistically significant differences between Group A and Group B with respect to gender distribution (*χ*^2^ =0.094, *p* > 0.05), cancer site (*χ*^2^ =2.622, *p* > 0.05), or TNM staging (*χ*^2^ =0.543, *p* > 0.05). These findings indicate that both groups were comparable at baseline, minimising the potential influence of treatment type and disease severity on the study outcomes.

**Table 2:** Characteristics of participants.

** ** **Characteristics**		**Group A** **(*n* = 35)**	**Group B** **(*n* = 35)**	***p***
**Age, Mean ± SD**		52.97 ± 7.59	55.52 ± 9.90	0.108^a^
**Gender** **(n, %)**	**Female**	6 ** (**17.14%)	7 ** (**10.34%)	0.759^b^
	**Male**	29 ** (**82.86%)	28 ** (**89.66%)	
**Cancer stage (n, %)**	**III**	14 ** (**40.00%)	15 ** (**51.72%)	0.762^b^
	**IVA**	11 ** (**31.43%)	10 ** (**24.14%)	
	**IVB**	10 ** (**28.57%)	10 ** (**24.14%)	
**Cancer site** **(n, %)**	**Buccal mucosa**	0 ** (**0.00%)	1 ** (**3.45%)	0.454^b^
	**Base of Tongue**	1 ** (**2.86%)	4 ** (**13.79%)	
	**Cricoid**	1**(**2.86%)	0 ** (**0.00%)	
	**Floor of mouth**	1**(**2.86%)	0 ** (**0.00%)	
	**Glottis**	0 ** (**0.00%)	1 ** (**3.45%)	
	**Hard palate**	0 ** (**0.00%)	1 ** (**3.45%)	
	**Larynx**	5 (14.29%)	3 ** (**10.34%)	
	**Left Buccal Mucosa**	5 ** (**14.29%)	1 ** (**3.45%)	
	**Metastasis of unknown origin neck**	1 ** (**2.86%)	0 ** (**0.00%)	
	**Nasopharynx**	2 ** (**5.71%)	0 ** (**0.00%)	
	**Oropharynx**	4 ** (**11.43%)	1 ** (**3.45%)	
	**Parotid**	0 ** (**0.00%)	1 ** (**3.45%)	
	**Retro molar trigone**	0 ** (**0.00%)	3 (10.34%)	
	**Right alveolus**	0 ** (**0.00%)	1 ** (**3.45%)	
	**Right Buccal Mucosa**	5 ** (**14.29%)	4 ** (**13.79%)	
	**Supraglottic**	2 ** (**5.71%)	1 ** (**3.45%)	
	**Tongue**	8 ** (**22.86%)	6 ** (**20.69%)	
	**Tonsil**	0 ** (**0.00%)	1 ** (**3.45%)	

**Notes.**

Values are presented as mean ± SD or frequency (%).

a Mann–Whitney U Test.

b Chi-square test.

Group A Exercise Oncology group Group B Control group n number of participants *p* Level of Significance SD Standard Deviation

The descriptive characteristics of PSQI and urinary melatonin for Groups A and B are presented in Table 3. For PSQI, groups did not differ significantly at baseline (*p* = 0.064). However, normality assumptions were partly violated (Tables 1 and 3). Mixed ANOVA showed a very strong Time × Group interaction (*p* < 0.001, *ω*^2^ =0.492). While the control group worsened slightly, the intervention group improved dramatically (Table 4). *Post hoc* tests confirmed that post-intervention PSQI was significantly better in the intervention group than in controls (large effect sizes, Cohen’s *d* > 2; Table 5). Although some violations of normality and heterogeneity were noted, the large and consistent effect sizes strongly support the robustness of the finding. The robustness of parametric tests under the Central Limit Theorem, particularly when homogeneity of variances is maintained, further supports the validity of the applied mixed ANOVA model. From an oncological perspective, heterogeneity in responses is expected due to treatment history, comorbidities, or medication use, and such deviations may reflect the clinical reality rather than methodological flaws.

**Table 3:** Descriptive statistics for PSQI and melatonin.

**Outcome**	**Time**	**Group**	**Pre Mean (SD)**	**Shapiro–Wilk**	***p***
**PSQI**	Pre	Group A	13.97 (2.30)	0.886	0.002
		Group B	13.06 (1.73)	0.932	0.031
	Post	Group A	7.09 (1.15)	0.925	0.020
		Group B	14.14 (2.64)	0.810	<0.001
**Melatonin (ng/L)**	Pre	Group A	285.42 (87.53)	0.852	<0.001
		Group B	363.67 (93.82)	0.784	<0.001
	Post	Group A	369.75 (92.95)	0.931	0.030
		Group B	305.88 (92.11)	0.860	<0.001

**Notes.**

Group A Exercise Oncology group Group B Control group ng/L nanograms per litre *p* level of significance PSQI Pittsburgh Sleep Quality Index SD Standard Deviation

**Table 4:** Mixed analysis of variance for pittsburgh sleep quality index.

**Effect**	**Sum of squares**	**df**	**Mean square**	**F**	***p***	*ω* ^2^
Time (RM Factor 1)	6,166	1	6,166	1.247	0.268	0.001
Group	1,811	1	1,811	0.153	0.697	0.000
Time x Group	176,715	1	176,715	35.725	<0.001	0.129

**Notes.**

df degrees of freedom F F statistic *p* level of significance RM Repeated Measures *ω*^2^ omega squared (effect size)

**Table 5:** *Post hoc* comparisons for Pittsburgh sleep quality index and urinary melatonin.

**Outcome**	**Comparison**	**MD**	**95% CI for MD** **(Lower, Upper)**	**SE**	**t**	**Cohen’s** ***d***	**95% CI for** ***d*** **(Lower, Upper)**	***p***
**PSQI**	Pre v/s Post (Group A)	−84.33	(−130.02, −38.64)	16.81	−5.07	−0.92	(−1.46, −0.38)	<0.001
	Pre v/s Post (Group B)	57.78	(12.09, 103.47)	16.81	3.44	0.63	(0.111, 1.15)	0.004
**Urinary** **Melatonin**	Group A v/s Group B	−96.89	(−141.5, −52.31)	22.33	−4.34	−1.13	(−1.69, −0.58)	<0.001

**Notes.**

CI Confidence Interval Cohen’s *d* effect size Group A Exercise Oncology group Group B Control group MD Mean Difference *p* level of Significance PSQI Pittsburgh Sleep Quality Index SE Standard Error

PSQI comparisons were adjusted using Holm–Bonferroni correction; melatonin comparison was adjusted using Tukey’s Honestly Significant Difference test.

Descriptive statistics showed that baseline melatonin values differed between groups (*p* < 0.001; Table 1). Independent *t*-tests and Mann–Whitney tests at baseline indicated significant group differences, with higher melatonin in controls. ANCOVA revealed that baseline melatonin level was a strong predictor (*p* < 0.001), but the group effect was not significant (*p* = 0.372; Table 6). This suggests that, after adjusting for baseline, there was no independent effect of the intervention on melatonin. *Post hoc* comparisons still showed raw mean differences, but these likely reflect baseline imbalances rather than a treatment effect (Table 5).

**Table 6:** Analysis of covariance for urinary melatonin.

**Effect**	**Sum of squares**	**df**	**Mean square**	**F**	***p***	*ω* ^2^
Group	5,916	1	5,916	0.809	0.372	0.000
Baseline (covariate)	99,236	1	99,236	13.565	<0.001	0.154
Group x Baseline	1,007	1	1,007	0.138	0.712	0.000

**Notes.**

df degrees of freedom F F statistic p level of significance *ω*^2^ omega squared (effect size)

**Figure 2:**
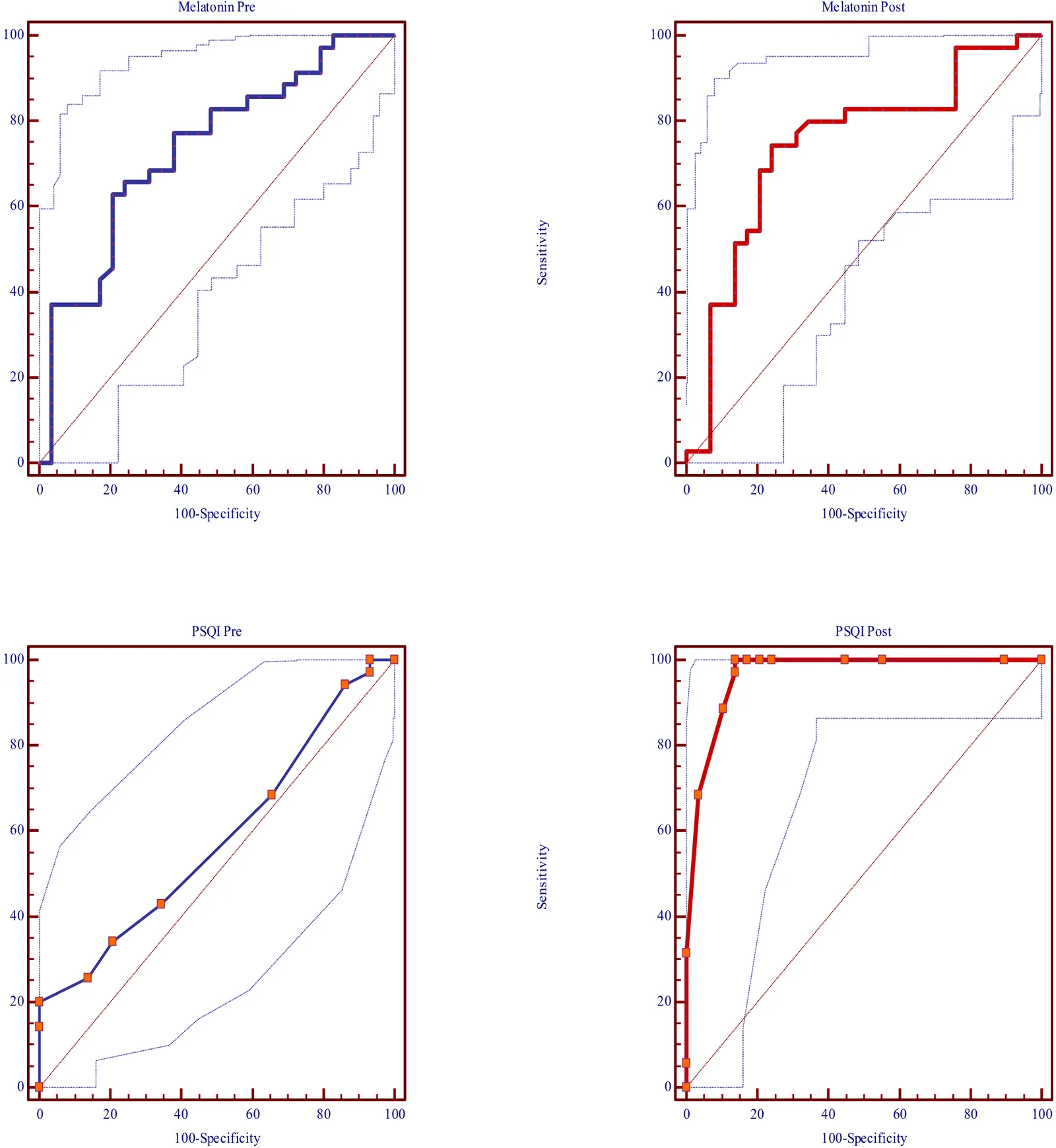
Receiver Operating Characteristics (ROC) curve analysis of pittsburgh sleep quality index score and melatonin in head and neck cancer patients.

ROC curve analysis, shown in Tables 7 and 8, demonstrated that PSQI Pre had an AUC of 0.582 (*p* = 0.251), indicating lower discriminatory ability, while PSQI Post displayed a remarkably high AUC of 0.966 (*p* < 0.0001), signifying excellent diagnostic accuracy for evaluating sleep quality. ROC curve analysis, shown in Tables 5 and 6 and Fig. 2, revealed promising results, with both melatonin pre and melatonin post demonstrating significant AUC values of 0.734 (*p* = 0.0002) and 0.742 (*p* = 0.0002), respectively, indicating potential as useful diagnostic markers for assessing circadian rhythm. Additionally, cut-off values were determined for each parameter. For the PSQI Pre, a cut-off value of >16 yielded a sensitivity of 20% (95% CI [8.4–36.9]), displaying a high specificity of 100% (95% CI [88.1–100.0]). PSQI Post, with a cut-off value of ≤10, exhibited a sensitivity of 100% (95% CI [90.0–100.0]) with specificity at 86.21% (95% CI [68.3–96.1]). For melatonin pre, a threshold of ≤332.547 resulted in a sensitivity of 62.86% (95% CI [44.9–78.5]) and a specificity of 79.31% (95% CI [60.3–92.0]). In contrast, for melatonin post, with a cut-off value of > 332.547, sensitivity increased to 74.29% (95% CI [56.7–87.5]) while specificity was 75.86% (95% CI [56.5–89.7]). These results indicate that both melatonin pre and melatonin post scores demonstrate diagnostic potential, while PSQI post scores show excellent diagnostic accuracy for distinguishing between good and poor sleep quality in these patients.

**Table 7:** Receiver operating characteristics curve analysis of outcomes.

	**Area under the ** **ROC curve ** **(AUC)**	**Standard error**	**95% CI**	***p*** ** value**
PSQI Pre	0.582	0.0713	0.452 to 0.704	0.2514
PSQI Post	0.966	0.0216	0.887 to 0.995	<0.0001^*^
Melatonin Pre (ng/L)	0.734	0.0631	0.609 to 0.837	0.0002^a^
Melatonin Post (ng/L)	0.742	0.0647	0.618 to 0.844	0.0002^a^

**Notes.**

* Significant at 0.0001 level.

a Significant at 0.001 level.

AUC Area Under Curve CI Confidence Interval ng/L nanograms per litre ROC Receiver Operating Characteristics *p* level of significance PSQI Pittsburgh Sleep Quality Index

**Table 8:** Cut off values, sensitivity and specificity for outcomes.

** Outcome**	**Cut off value**	**Sensitivity**	**95% CI**	**Specificity**	**95% CI**
PSQI Pre	>16	20	8.4–36.9	100	88.1–100.0
PSQI Post	≤10	100	90.0–100.0	86.21	68.3–96.1
Melatonin Pre (ng/L)	≤332.547	62.86	44.9–78.5	79.31	60.3–92.0
Melatonin Post (ng/L)	>332.547	74.29	56.7–87.5	75.86	56.5–89.7

**Notes.**

CI Confidence Interval ng/L nanograms per litre PSQI Pittsburgh Sleep Quality Index

## Discussion

This study evaluated the impact of an exercise oncology program on sleep quality and circadian rhythm in patients with HNC undergoing chemo-radiation therapy. Although the benefits of physical activity on sleep and quality of life in cancer populations are well documented, most available evidence is derived from heterogeneous cancer cohorts or post-treatment survivors. Data focusing specifically on patients with HNC undergoing active chemo-radiation therapy, particularly integrating both sleep quality and circadian biomarkers, remains limited. The present study, therefore, provides clinically relevant, population-specific evidence from a low- and middle-income country setting during a highly vulnerable phase of treatment, where healthcare delivery, nutritional status, and supportive care resources may differ substantially from high-income contexts.

The findings highlight a clear benefit of exercise in improving subjective sleep quality, with patients in the intervention group reporting marked reductions in sleep disturbances compared to controls. In contrast, urinary melatonin levels did not demonstrate a significant change attributable to the intervention, despite observable differences in raw values. Taken together, these findings indicate that exercise may serve as a supportive strategy to improve sleep quality during cancer treatment, although its influence on circadian biomarkers such as melatonin remains inconclusive.

The intervention was clearly associated with an improvement in subjective sleep quality but not with measurable alterations in melatonin secretion after adjusting for baseline values. This dissociation suggests that the positive effects of exercise on sleep in patients with HNC may operate through mechanisms other than circadian regulation, for example, enhanced physical fitness, reduced treatment-related fatigue, or improvements in psychological well-being.

Patients with HNC face considerable challenges due to complex treatment regimens and their associated side effects, both of which can significantly impair sleep quality and disrupt circadian rhythm (Pecorari et al., 2024). Poor sleep quality is common in this population, compounding the burden of the disease and its treatment (Pecorari et al., 2024; Chen et al., 2024). The marked decline in melatonin levels by the third week points to an early onset of circadian rhythm disruption as an adverse effect of chemo-radiation therapy (Yeh, 2010; Kartini et al., 2020; Amidi & Wu, 2022). Sleep disturbances in these patients may arise not only from direct treatment effects but also from the combined physical and psychological stressors of the illness (Li et al., 2023; Amaral et al., 2022).

Inadequate sleep contributes to fatigue, mood disturbances, cognitive decline and overall reductions in quality of life (Zhao et al., 2016). Moreover, the consequences of sleep disturbances may persist after treatment ends, resulting in chronic sleep issues and persistent impairment of day-to-day functioning (Anjanappa et al., 2020; Kok et al., 2022). Therefore, addressing sleep quality and circadian rhythm regulation is essential for improving the overall quality of life and optimizing rehabilitation and treatment outcomes for HNC patients.

This randomized controlled trial aimed to investigate how an exercise oncology program influenced the quality of sleep and circadian rhythm in patients undergoing chemo-radiation therapy for HNC, specifically focusing on urine melatonin excretion. Melatonin, a key regulator of the circadian rhythm, not only plays a crucial role in sleep, but also serves as a reliable marker of overall sleep quality *via* its excretion levels (Samuel et al., 2019; Avancini et al., 2023). The results showed that the exercise oncology group demonstrated improved outcomes compared to the control group, underscoring the influence of various factors, including physical activity, on circadian rhythm and sleep patterns, as shown in prior research by Gururaj et al. (2021). Over the course of seven weeks, that study observed a decrease in melatonin levels, sleep duration, and physical activity. They also observed a strong negative correlation between physical activity and sleep efficiency, a moderate relationship between sleep efficiency, total sleep time, and step count, and a weaker relationship with disruption of the circadian rhythm. High-level evidence from a recent systematic review and meta-analysis demonstrated that exercise-based interventions significantly improve sleep-related outcomes and circadian rhythm parameters across cancer populations (Gururaj et al., 2024). These results highlight the relationship between melatonin levels, the quality of sleep, and the possible advantages of exercise oncology program in re-establishing a more typical circadian rhythm (Pérez et al., 2023).

Regular exercise not only enhances melatonin levels, but also stimulates the release of endorphins, which naturally elevate mood, while decreasing the levels of stress hormones like cortisol (Satish et al., 2023). This helps alleviate symptoms of fatigue and improve mood, contributing to a higher quality of life. Additionally, the cardiovascular advantages of physical activity, including better heart and lung function, can enhance endurance and energy levels, which may be compromised during cancer treatment (Mustian et al., 2013).

Exercise has been linked to better immune function and improved ability to tolerate treatment-related side effects. Incorporating exercise into their routine could help HNC patients experience physical benefits as well as mental and emotional resilience during their treatment process. Thus, exercise serves as a multifaceted approach to support both physical strength and overall well-being in individuals undergoing treatment for HNC (Lyu et al., 2024).

Beyond structured exercise programs, physical activities such as yoga, tai chi, and walking have also been shown to improve sleep outcomes (Lyu et al., 2024; Li et al., 2024; Song et al., 2025; Samuel et al., 2015). In a randomized controlled trial involving 410 cancer survivors, a standardized yoga intervention significantly improved global sleep quality, sleep efficiency, and sleep medication usage compared to standard care (Li et al., 2024). Tai chi has similarly demonstrated consistent benefits; a meta-analysis in older adults revealed that tai chi significantly reduced PSQI scores, indicating better overall sleep quality (Li et al., 2024; Song et al., 2025). An analysis of 34 RCTs involving 3,083 breast cancer survivors found that walking significantly improved sleep quality and was recommended for cancer survivors as a safe, accessible form of physical activity (Samuel et al., 2015). Incorporating these modalities in addition to traditional exercise may offer more patient-centered, feasible options for managing sleep disturbances in HNC patients.

This study had several limitations that should be considered when interpreting the results. First, the small sample size may have influenced the generalization and statistical power of the findings, limiting the ability to draw broader conclusions. Blinding of participants to the intervention was not feasible due to the nature of exercise-based programs, may possibly introducing performance bias. The same investigator delivered the intervention and conducted the outcome assessments. While these may introduce a risk of bias, efforts were made to mitigate this using standardized protocols and validated, objective outcome measures, including the PSQI and urinary melatonin assays. Melatonin levels, although measured objectively, did not show a significant treatment-related change after adjusting for baseline differences. These findings raise the possibility that melatonin secretion may be shaped by factors beyond the intervention, including medication use, overall disease burden, or circadian disruption associated with cancer progression. Another limitation of the study was the absence of blinding of the investigator to group assignments, which could have introduced bias in outcome assessment and interpretation. In addition, subgroup analyses across different stages of cancer were not performed. Such comparisons could have yielded further insight into whether disease stage modifies the effect of the intervention.

Looking ahead, future research could broaden the scope by including patients with other cancer types to assess whether the benefits of exercise oncology programs on sleep quality and circadian rhythm are consistent across populations. Larger sample sizes would improve statistical power and strengthen conclusions, particularly with respect to circadian biomarkers such as melatonin. Incorporating additional objective measures of circadian rhythm, such as repeated melatonin sampling or actigraphy, may also provide more sensitive information about the biological mechanisms driving improvements in sleep. Furthermore, the use of advanced and objective tools, including validated sleep analyzers, could enhance the accuracy and reliability of sleep assessment, thereby adding greater rigor to future studies.

## Conclusion

This study suggests that an exercise oncology program may improve sleep quality in patients with HNC undergoing chemo-radiation therapy. The results also point to urinary melatonin as a potential biomarker for circadian rhythm assessment in this population, although the intervention itself does not show an independent effect on melatonin secretion after adjustment for baseline values. If confirmed by further studies, these findings underscore the value of incorporating structured exercise programs into the comprehensive management of HNC patients as a means of supporting healthier sleep. This in turn can help refine rehabilitation strategies to enhance sleep quality and to further clarify the role of circadian rhythm regulation in this vulnerable group.

## Supplemental Information

10.7717/peerj.21409/supp-1 Supplemental Information 1 CONSORT Checklist

10.7717/peerj.21409/supp-2 Supplemental Information 2 Dataset

10.7717/peerj.21409/supp-3 Supplemental Information 3 Code book for TNM staging Code book to convert numbers of TNM staging to their respective factors

## Additional Information and Declarations

### Data Availability

The following information was supplied regarding data availability: The raw data are available in the Supplemental File.

### Clinical Trial Registration

The following information was supplied regarding Clinical Trial registration: CTRI/2023/11/059839
