# Supplementary material for: Efficacy of an exercise oncology program on sleep quality and circadian rhythm in head and neck cancer patients undergoing chemo-radiation therapy: a randomized controlled trial
Source: PeerJ. 2026 Jun 25;14:e21409. doi: 10.7717/peerj.21409 (PMC13310484; doi:10.7717/peerj.21409)
Supplement: Supplemental Information 3 — Code book to convert numbers of TNM staging to their respective factors [file peerj-14-21409-s003.docx]

**TNM Staging** [Deschler, Daniel. (1991). Neck Dissection Classification and TNM Staging of Head and Neck Cancer.]

**Stage Grouping**

|  | Tumour | Nodes | Metastasis |
| --- | --- | --- | --- |
| Stage 0 | Tis | N0 | M0 |
| Stage I | T1 | N0 | M0 |
| Stage II | T2 | N0 | M0 |
| Stage III | T3 | N0 | M0 |
|  | T1 | N1 | M0 |
|  | T2 | N1 | M0 |
|  | T3 | N1 | M0 |
| Stage IVA | T4a | N0 | M0 |
|  | T4a | N1 | M0 |
|  | T1 | N2 | M0 |
|  | T2 | N2 | M0 |
|  | T3 | N2 | M0 |
|  | T4a | N2 | M0 |
| Stage IVB | T4b | Any N | M0 |
|  | Any T | N3 | M0 |
| Stage IVC | Any T | Any N | M1 |

Tis: Carcinoma in situ

T1: The tumour is 2 cm or smaller and confined to the site of origin

**T2**: The tumour is larger than 2 cm but 4 cm or smaller, still confined to the site of origin.

T3: The tumour is larger than 4 cm or has grown into surrounding structures but remains confined to the head and neck region.

T4a: The tumour has invaded adjacent structures such as the jawbone, skin, or other vital structures within the head and neck region.

T4b: The tumour has invaded more critical structures such as the base of the skull or major arteries (like the carotid artery).

**Any T**: The tumour may be of any size and may have spread to adjacent structures.

**N0**: No regional lymph node involvement.

**N1**: The tumour has spread to a single regional lymph node on the same side of the neck (and the node is 3 cm or less).

**N2**: Multiple lymph nodes involved, or a single node larger than 3 cm but equal to or less than 6 cm.

**N3**: Lymph node involvement larger than 6 cm.

**Any N**: The cancer can have spread to any regional lymph nodes.

M0: No distant metastasis.

M1: Distant metastasis is present (cancer has spread beyond the head and neck to other organs, such as the lungs, liver, or bones).
